# Australian Nurse Practitioner Practice: Value Adding through Clinical Reflexivity

**DOI:** 10.1155/2015/829593

**Published:** 2015-01-29

**Authors:** Michelle Woods, Giuliana Murfet

**Affiliations:** ^1^Royal Hobart Hospital, Diabetes Centre, 5th Floor, 70 Collins Street, Hobart, TAS 7000, Australia; ^2^School of Health Sciences, University of Tasmania, Hobart, TAS 7000, Australia; ^3^Diabetes Centre, Tasmanian Health Organisation-North West, P.O. Box 258, Burnie, TAS 7320, Australia

## Abstract

The role of the Australian Nurse Practitioner (NP) is in its infancy and at a crossroads where extensive research demonstrates effective quality care and yet the role remains underrecognised and underutilised. The translation of practice into “value” is critical for the sustainability of NP roles and requires the practitioner to adopt a systematic method of inquiry. Kim's (1999) “Critical Reflective Inquiry” (CRI) method was adapted by two Australian NPs who specialise in diabetes and chronic disease management. Kim highlights the intent of CRI as understanding the meaning of practice, delivering improvements to practice through self-reflection, and the critique of practice that can lead to practice changes and development of new models of care translated to “products” of value. Based on the thematically analysis of 3 years of CRI application, the authors formed 5 headings that represented the NP's practice as Specialised Care Access, Complications and Diagnostics Interventions, Pharmaceutical Treatment, Vulnerable Populations, and Leadership. The utility of CRI demonstrates how NP practice is integral to a continuous cycle of addressing health care services gaps, and the conversion of “products” into “value” and positions the NP to assimilate the role of the practitioner-researcher.

## 1. Introduction 

The Australian Health Care System has a fundamental focus on providing access to safe and most appropriate care for the person's health care needs in the timeliest manner [1]. As with most westernized countries, health care reform in Australia is warranted in response to demographic shifts resulting in an aging population and increasing prevalence in noncommunicable disease [2]. Furthermore, it is the comorbidity nature of chronic diseases with the increasing complexity in care needs of individuals and populations that has resulted in burgeoning health care expenditure [3]. In this context of increasing service needs, gaps in health care delivery an emphasis on providing timely efficient care that the Australian Nurse Practitioner (NP) roles have been developed. The development of NP roles has often been in response to health care issues involving limited and poor access to health services along with provision of services in marginalized groups across the three tiers (primary, secondary, and tertiary) of the health care system [1, 4, 5].

Over the past 15 years integration of the Australian NP roles has been established by key legislative, educational, and regulatory standards [6, 7]. Australian NP models of care to date demonstrate innovations to meet the changing health needs of populations, substantiated through evidence of efficacy, feasibility, safety, effectiveness, quality, and cost [8–10]. Research and leadership in clinical practice combined with new models of managing patient flow improving efficiencies in health resources and access to health services are key characteristics of the NP role [11–15].

Despite these fundamental changes and evidences, the full integration of the NP role has been slow and small in comparative numbers to the UK and USA. In Australia, the NP movement is in its infancy with numbers of authorized NPs reaching 1000 in 2013; in 2012 it was estimated that approximately 30–50% of authorized NP were working within NP roles [16]. This slow rate of integration and utilization of the full potential of NP practice positions the NP movement at crossroads where a research practice gap has emerged leaving an “untapped health care solution” [17].

Systematic and local barriers have been identified throughout the public and private health care sectors and within the tiers of health care delivery [1, 16]. Analysis as to why the integration has not been expedited points to the unsubstantiated economic reimbursement of NP services [1]. The majority of NPs are employed in secondary and tertiary care provision within public hospital and are renumerated through State Government salaries [16]. In the primary tier of health care, NP services are renumerated through the national Commonwealth Government Medicare Benefits Schedule (MBS) [18]. MBS provider numbers to this scheme are accessed by private NPs (predominately in primary care) but reimbursement is more than fifty percent less relative to general medical practitioners [18]. Consequently, this discrepancy in Commonwealth MBS reimbursement for NP services has resulted in unsustainable business models for NPs practicing in primary care.

Furthermore, NPs employed in the state and territory public sector do not have access to MBS provider numbers [19] unlike their medical colleagues. Thus, when compared to their medical colleagues working in the public hospital system, NPs do not have a direct economic value for the provision of the services they provide. This discrepancy allows medical colleagues to generate state hospital funding through Commonwealth reimbursable public outpatient services. This misalignment between funding models with NP models of care has created systematic barriers to integrate the NP role [1]. In addition, intra- and interprofessional dynamic tensions and subsequent misunderstanding of the nature of the NP role have led to difficulty in articulation and implementation of roles [16, 18, 20, 21].

At national level, the Australian College of Nurse Practitioners published a position paper “*The economic value and potential of Nurse Practitioners in Australia*” [1] highlighting the discrepancy in the feasibility of NP practice and role implementation. In a time of economic rationalization* this paper* has served to aid in lobbying the Commonwealth government, in particular, to address the barrier of service funding for NP practice necessary for sustainability of the current health care system. To date many fully qualified and authorized to practice NPs continue to be underutilized and underemployed [17].

At this point in time, in all health care arenas, an Australian NP must not only come to terms with indirect and/or unsubstantial reimbursement for services in the public and primary care sector respectively, but also overcome an organizations' reluctance to commit to funding NPs, who are more highly paid than other nurses [22]. Unlike the USA and UK, direct economic rationalization for an Australian NP is not substantiated [23–25]; the value of NP practice in addressing an ailing over burgeoning health care system falls on each Australian NP to demonstrate how they are “value adding” to a health care system.

It is not within the capacity of this paper to analysis further how to improve health lobbying and policy making to expedite funding models that support the viability of current and future NP practice. The intent of this paper is to highlight the necessary onus and opportunity each practicing NP has to articulate how their practice impacts patient outcomes and translates to “value adding” to the health care systems that they serve.

The Australian NP is well positioned and trained to address a high level of clinical and system complexities. In Australia, “Nurse Practitioner” is a legal title for an advanced practice nurse who has completed both advanced university study at Master degree level and extensive clinical training to expand upon the traditional role of a Registered Nurse [26]. The capacity to practice autonomously ensures a seamless clinical alignment in advanced physical assessment, order of diagnostic tests and their interpretation, initiation of referrals to relevant health care providers, and prescribing of appropriate medications and other therapies as needed [7].

Australian NP standards of practice require NPs to demonstrate four domains: clinical, education, research, and leadership. As primarily clinicians, education, research, and leadership domains are couched within each clinically focused standard. This framework reflects the NP capacity to perform in complex clinical scenarios as an expert clinician, while demonstrating the synthesis and application of best practice evidence and high quality education in working with patients, family members, and colleagues [27]. Research utilization is supported through evidence-based care and quality management; however, as primarily clinicians the capacity to conduct research is often unobtainable due to the prime work load focus of clinical care. Ironically, it is through the nature of advanced clinical practice where quality patient care and capacity to demonstrate “value adding” take place [12, 22, 28–31].

Australian NPs as clinicians and researchers have risen to this challenge and similar to international studies have identified that the NP role has a substantial contribution to meeting the needs of vulnerable populations and in the provision of high quality health care [8–15, 23]. Significant outcomes for vulnerable population have been identified in the provision of quality health care to older people and caring for people with dementia [12, 28] and meeting the needs of Australians living with poor mental health [29, 30]. Further studies have reviewed the necessary framework and integration strategies that are required for successful NP role implementation leading to improvements in health care system outcomes [31, 32]. 

The capacity to provide research on health care impact and efficacy of practitioners' roles often requires the partnership with academic nursing research teams supported by research and organizational funding [33]. However, for every Australian NP without a research team, determining the impact an NP has on patient care and/or the value of care measured through quasi-experimental and/or experimental and/or qualitative methodology is not feasible. Furthermore, hindering a practitioner's engagement in research is a common perception that research is conducted by a researcher as opposed to a practitioner-researcher [34, 35].

A predicament for the Australian NP is either to wait for high level policy decisions to be made to support direct remuneration for NP services or to integrate and demonstrate within their own practice arena how they “value add” to the health care system. The second alternative provides a means to influence and feedback to organization decision making for future investment and sustainability in NP roles. It is the opinion of the authors that sustainability of the Australian NP practice can only be realized when, in the absence of direct economic benefits, the impact of practice is transformed and articulated as “value” to the health care delivery system. This paper will demonstrate how the research methodology of Critical Reflection or Reflexivity [36, 37] is closely aligned to NP practice and provides a means to articulate how NP practice impacts patient care and is “value adding” to health care systems.

Ultimately, the act of reflection in practice is essential to professional integrity and delivery of responsive quality care. Reflection, as an entity of practice, is a purposeful review of ones actions through an in-depth consideration of events or situations outside of oneself [38]. For a practitioner, the practice becomes purposeful when the cognitive application of clinical reasoning skills, problem solving, and critical thinking in and on practice is evident [27, 39].

The intent of reflective practice (RP) is to apply a consistent and systematic approach to investigating and resolving problems, with ethical practice guided by professional discretion [36, 37]. This approach to practice has been adopted by nursing regulatory bodies, particularly in the UK and Australia, as a means for nurses to ensure professional accountability and practice development [40].

RP begins to move from transformational learning to transformational practice through the research methodology founded on Reflexivity or Critical Reflection [37]. At a basic level, reflexivity, synonymous with critical reflection, means turning back on oneself with an element of critic, analysis incorporated into the reflective process. The term critic is defined by the oxford dictionary “*as having a decisive importance in the success or failure of something crucial *[41].” What is implicit in this definition when related to practice is that knowledge attained in understanding discrepancies in service delivery and/or quality of care leads to the onus to be with or change a circumstance.

It is this critical component that leads to the notion of transformational practice that embodies a commitment to challenge dominant norms and assumptions [37]. Freshwater and Rolfe explain that critical reflection requires a practitioner to consider the social and political context in which practice takes place and that further action may result in “resisting power and constraints and to open up to new theoretical and practical possibilities [37].” Thus, reflexivity goes beyond the personal practice of self-examination.

The practice implication of reflexivity is not only to translate evidence into practice but also to translate practice into evidence. As a reciprocal notion to research applied to practice, Freshwater and Rolfe [37] introduce the methodology of reflexivity as a means to convert practice reflection into research. In this way, the practitioner embedded in practice takes on the role of a “practitioner-researcher.” The role requires the practitioner to integrate both practice and research through an inductive lens in the exploration of practice.

There are a number of challenges this position has as a means for knowledge generation and acquisition [42]. Initially, the immersed position of the practitioner's insider perspective challenges the dominate paradigm of both a technical rationalist model where a researcher comes in from outside to conduct objective research on practitioner(s)/patients. In addition, a practitioner-researcher utilizing reflexivity is empowered to develop and apply contextual and local knowledge as opposed to the traditional positivist deductive paradigm of knowing exactly what something is prior to application [37].

## 2. Method: Critical Reflection Inquiry (CRI)

The intent in utilizing CRI is to demonstrate how the practice of NP results in “products” [43] that can be conveyed and converted into value to the health care systems that they serve (Table 1). The translation of practice into value calls for a systematic method of inquiry that leads to legitimacy in not only the process of practice but also ultimately the impact and outputs of NP practice. As a method of reflexivity, CRI provides a systematic approach where practitioners synthesises knowledge for its application to practice [43] and results in “products” of impact. Kim highlights the intent of CRI as 3-fold in aiding in understanding the meaning of practice and delivering improvements to practice through self-reflection and the critique of practice that can lead to practice changes and development of new models of care translated to “products” of value [44]. The significance in this method is the transformation of practitioner reflective knowledge in and on practice; leading to critically oriented change and is shared in the professional domain.

Kim's [44] CRI method is congruent with the Australian NP standards of care [27] and the identified need for NPs in Australia to generate knowledge of* how* they are “value adding” to their specific health care systems [16]. Kim states (p.1205) that her method of inquiry “is founded upon the ideas in action science and reflective practice and critical philosophy,” resonating with practitioner's intent to transform practice knowledge into critically oriented change [44].

Although there is no published evidence of CRI utilization for NPs, CRI as a method has the potential to guide the NP to assimilate the role of the practitioner-researcher. This requires the NP to move beyond RP as integral to their own practice to also demonstrate* how* they impact and transform their practice in their practice arena. Essentially, this application of method has the potential to articular NP innovation that can be further shared in the development of corporative inquiry with nursing researchers [45] and policy makers [44].

**Figure 1:**
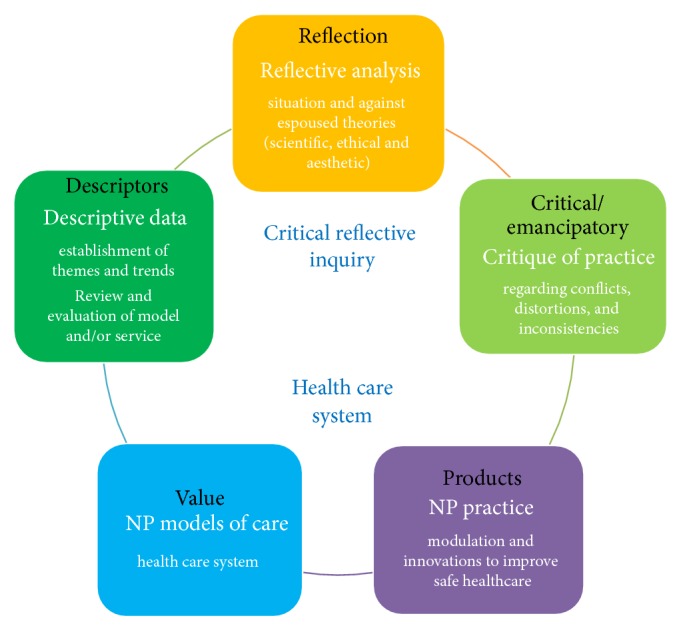
Model of Critical Reflective Inquiry supporting NP practice.

Kim's CRI method requires the practitioner to engage in 3 phases (Figure 1); the descriptive phase, the reflective phase, and the critical/emancipatory phase represent the processes within the method [44]. Each of the three phases is associated with “products” as follows:

- Descriptive phase: descriptive narratives, and* reflexive data collection including case studies and clinical audits*.
- Reflective phase: examination of personal beliefs, assumptions, and knowledge aligned and tested with existing scientific knowledge analyzed in terms of ethical or valued standards.
- Critical/emancipatory phase: oriented towards changing practice through recognition of discrepancies in practice and best practice, a critique of distortions, inconsistencies, and incongruences between values/beliefs and practice.

For the purposes of this paper, Kim's [44] CRI method was adapted as an integral component of practice of 2 Australian NP specializing in diabetes and chronic disease management. Both authors are employed by the same public health care organization but practice in different geographical regions; a under served rural community and the other at a metropolitan secondary care center attached to a tertiary medical center. The tier of health care delivery is secondary care where referrals to speciality care centers are generated predominately by general medical practitioners. The NP's practices are autonomous and importantly work collaboratively with a multidisciplinary team consisting of specialized nursing, endocrinologist, and allied health professionals.

The combination of the CRI process integrated into an NP's practice supported by prompting questions (Table 1) processes and analysis has the potential to enable an NP to articulate the value that their practice has (Figure 1). As a method of reflexivity, CRI orientation is not to verify or refute hypotheses but rather to generate products of knowledge from practice episodes [35]. In addition, reflexivity methodology does not seek to govern multiple variables and their consequential interactions with practice but rather demonstrate practice transformation responsive to complexity of practice scenarios.

Mantzoukas [46] argues that reflexivity cannot be divorced from research biases and in fact the methodology is steeped in research biases (assumption and beliefs) that are integral to the research process. Validity lies in the transparency of the “researcher's bias is fully incorporated and becomes transparent throughout the study [46]”. Rigour of CRI method is revealed through the standardization of the research process responsive to the descriptive phase involving contextual writing, a reflective phase examining, contrasting narratives of practice with one's beliefs and assumptions (revelation of the researchers biases), and synthesis of knowledge (scientific, ethical, and aesthetic) [43]. It is this systematic process that provides self-emancipation to address insights into future assimilation of new innovations emerging from practice but may also bring result in an emancipatory culture in clinical settings [44, 47].

Although Kim's [44] method calls for a partnership between practitioner and researcher described as “a guide confident in participatory critical inquiry,” the authors identified and endorsed the concept of “practitioner-researcher” for the application of CRI. Partnership with academia research has and would be sought for further explorations of key findings identified through the adaptation of Kim's method.

## 3. Results and Discussion 

Over the course of 3 years the 2 NP formed a peer mentoring relationship [48]. The relationship provided each NP with the support necessary to step into and practice in a medical domain with a foundational nursing view. In the clinical practice arena the application of advanced practice nursing expertise and skills (in addition to extended practice of advanced assessment, differential diagnosis, investigative diagnostics, pharmacological management, prescribing, and monitoring) presented the NPs, an inductive view of practice across both a nursing and medical domain.

Multiple patient and practice episodes were generated over a 3-year period and analyzed through the form of content analysis for themes and patterns developed by Luborsky [49]. This means of thematic analysis lead to 5 thematic headings representing the NP's practice application: Specialised Care Access, Diagnostic and Complications, Pharmaceutical Treatment, Vulnerable Populations, and Leadership. Under each heading exemplars of the CRI process serve to illustrate how this method resulted in “products” that could be converted to “value” (Table 2).

### 3.1. Specialised Care Access (Points of Access and Models of Care)

#### 3.1.1. Exemplar 1

*Descriptive*. People living with complex type 2, type 1, or gestational diabetes are referred to speciality care. Predominately type 2 diabetes is treatable in primary care settings; however, the large increase in its incidence and complexity due to multiple morbidities increases the volume of patients referred to speciality clinics [50]. Hence, in the public health care system there is often up to a 6-month waiting time to see an endocrinologist; sequential follow-up in time-dependent medications can be stalled. In the case of an acute complication of diabetes, urgent treatment may also be necessary and access to speciality care in an outpatient setting is limited [50]. This can result in patients transfer to emergency department for treatments and medication adjustments that can be addressed in a speciality outpatient setting.

*Reflection*. The alignment of NP expertise and capacity has the potential to divert preventable emergency department and/or hospital admission. Potentially preventable hospitalizations (PPH) are defined as admissions that might ordinarily have been controlled or avoided [51]. PPH include selected chronic conditions and acute medical and adverse drug events.

*Practice Scenario*. 32-year-old female was identified as “poorly controlled diabetes” following assessment by NP. Patients phenotype and overt symptomology lead to the suspicion that the patients treatment of type 2 diabetes had not been adequately assessed and treatment on oral antidiabetic mediation was contributing to the poor control. Further, serum assessment revealed that the patient was in mild ketoacidosis; later NP initiated serum antibody testing confirmed that the patient was misdiagnosed and living with type 1 diabetes. Pending antibody screening the patient was started on insulin, had adequate support for medication titration, and adjusted well to new regimen. Potentially, this patient could have become very ill and admitted to the emergency department. Patient relieved with the diagnosis, hyperglycaemic symptomology resolved over the course of one week, and she was added to a type 1 diabetes clinical pathway.

*Critical/Emancipatory Phase*. An NP initiated weekly rapid response clinic was developed with the intent of assessing and treating patients newly diagnosed and/or poorly controlled. Over the course of 2 years this clinic has provided a rapid access service for GP referrals, in particular, in supporting patients who need prompt or immediate attention in addressing metabolic abnormalities and/or hyperglycaemic states contributing to chronic morbidities (diabetes foot ulcers, reoccurring urinary tract infections) and/or impending acute exacerbations of other morbidities (rheumatoid arthritis).

#### 3.1.2. Exemplar 2

*Reflection*. Large patient volume to the service increases the time interval to be reviewed by a specialist and follow-up of time-dependent therapies [50]. The NP's expertise in advanced assessment, therapies, and management of pharmacotherapeutics positions the NP to work collaborative with specialists to review patients at initial reviews or as follow-ups to speciality service [27]. In addition, the nursing focus adds a broad attention to addressing an array of complex issues that may be impeding a patient's diabetes self-management.

*Practice Scenario*. 53-year-old male is living with complex mental health history and diabetes. Poorly controlled diabetes and his sustained hyperglycemia have led to progressive organ disease and neuropathies. The chronic pain, nocturia, and disruptive sleep resulted in poor reserves to aid mental illness. Patient was referred to NP care. Focus was in partnership with GP in addressing patient's greatest fears and loss of vision and independence. In building trust with the patient, misconceptions about his diabetes control could be addressed. This resulted in change in medication regimen and he was connected with services to aid in his vision impairment. The negative spiral effect of sustained hyperglycemia on his capacity to function with daily activities of living was reinforced. Prior to the NP consistent care approach the patient had incurred 7 hospital admissions in 12 months. Over the course of 12 months his glycaemic control was reduced by 50% and he had a 100% reduction in hospital admissions. 

*Critical/Emancipatory Phase*. The above scenario is one of many scenarios that substantiated an NP led clinic in consistent care for patients referred by diabetes services and/or specialist that require a fixed term consistent care focus. In addition, NP clinics were coordinated with the clinic nurse to actively decrease waiting and follow-up times for patient reviewed by specialists. In 2012, a 16-question patient survey of the Quality of Care provided by an NP was conducted. Return rate was 60% and when asked “overall how would you rate the care provided by the NP,” 58% responded excellent and 36% very good. To support this process, in 2014 an NP conducted Clinical Audit (3-month) was undertaken reviewing patient referrals categorized as “nonurgent.” The assessment and verification of these referrals from primary care to secondary care verified that there is a need to support primary care practices. This was in the areas of knowledge gaps and/or resource aid in adjustment of time-dependent medications, such as insulin. Further actions to address supporting primary services for the care of people living with diabetes are discussed under the* Diabetes and Leadership* thematic heading.

### 3.2. Diagnostics and Complications

#### 3.2.1. Exemplar 1

*Description*. Most patients living with diabetes referred to secondary care live with multiple morbidities. The interplay between diabetes and multiple morbidities can be a bidirectional relationship between poor glycaemic control and exacerbation of morbidities [52]. The nature of diabetes as a vascular disease affects every organ in the body [53]. Often a comprehensive assessment requires breadth and depth in the assessment of physiological systems and psychosocial attributes [54]. An NP specialized in this area assesses the complexity of diabetes management, both as a disease state and the persons narrative of living with diabetes. The combination of a thorough review, capacity to utilize differential diagnosis, and initiation and interpretation of investigative tests allows for comprehensive care [27]. In addition, there is an element of care coordination in the NP's role as patients living with chronic complication have multiple providers [54].

*Reflection*. People living with long standing type 1 diabetes can have erratic daily glycaemic levels with significant unexpected hypoglycemia [54, 55]. This has a considerable impact on a person's livelihood and daily function [55]. The specialist analysis of daily blood glucose levels can reveal a myriad of possibilities for erratic control [54]. One exemplar of the “value” of an NP's comprehensive advanced assessment is through the analysis of blood glucose levels that indicated a number of patients living with undetected gastroparesis.

*Practice Scenario*. 27-year-old female with diabetes of 22 years duration is living with diabetes and microvascular comorbidities. Previous long term above target glycaemic control in youth had led to neuropathies and retinopathy. The patient was referred to the NP and the focus was on working in partnership with the person to achieve best possible outcomes through assessment and enquiry. Data generated reveals the NP collects information on not only diabetes management and activity but also eating habits (time, frequency, “if there is anything that disrupts eating”) and gastrointestinal sensation/symptoms during and after meals and overnight (common patient narratives in this case and others, “feel like food is stuck in my throat often,” “I can wake up bloated in the morning,” and “I always have indigestion after meals and my stomach is often bloated”). The patient reported that this generally upper epigastric discomfort had been long standing, potentially over two years. Discrepancies are noted between patterns of BGLs and low BGLs. These low BGLs throughout the day were not consistent with meals or activities at times they occurred directly following a meal. Diabetes control initially improved with intensive therapy to prevent false positive investigation; a gastric emptying study was then ordered via endocrinologist (as not permitted by NP). The highly positive result was then treated with antiemetic medication, education, and dietary changes by the NP. Following these steps the young lady had a reduction in her HbA1c by 1.7% and reduction in hypoglycemic events and reported an improved quality of life. Epigastric pain and nausea were resolved and hypoglycemic episodes did not occur in the workplace.

*Critical/Emancipatory Phase*. Over the course of 12 months in collaboration with her endocrinologist colleague, the NP screened 12 patients for gastroparesis with a 100% positive result. The NP worked with these patients in a pharmacological and nonpharmacological treatment plan involving the use of antiemetic's and/or meal plan with more small frequent meals lower in fat and fiber and referral to dietician. In further assessment, all patients diagnosed with gastroparesis and under the care of the NP voiced reduction in gastric symptoms and hypoglycemic episodes and an improved feeling of wellbeing.

The reflection* in and on* comprehensive assessments and identification of diabetes related complications has led to numerous NP critically orientated changes. The holistic nursing perspective of the interplay between chronic conditions and the persons capacity to living with and manage chronic morbidities calls for an analysis of risks to a patient's health, wellbeing, and independence [55]. NP interventions extend beyond the focus of the primary index disease. For example, the interplay between hyperglycemia, peripheral neuropathy, nocturia, and falls risk is substantial for aging people with diabetes with poor glycaemic control [52]. An NP engaged in critically oriented change requires the NP to extend beyond treating the hyperglycemia and to also incorporate interventions such as advocating for and building professional networks for community resources, for example, falls risk preventions classes.

### 3.3. Pharmaceutical Access (Box 1) 

#### 3.3.1. Exemplar 1

*Description*. Often patients will need up to 15 different classes of medications when living with diabetes and for the treatments of diabetes complications [56]. There are a number of issues for patients presenting with an extended medication list. These include but are not limited to poor knowledge and understanding of indication; dosing and use of medications; prohibitive cost of medications; and potential medication interactions [56]. For the NP a patient consult is an opportunistic time to review a patient's beliefs, understanding, and medication dosing schedule and also any concerns regarding medication. In addition, as a specialist NP there is the role of assessing the adequacy, effectiveness, and evidence based rationale for the patient's medications [27].

*Reflection*

*Practice Scenario*. 67-year-old female lived with diabetes for 16 years. The patient was referred to specialty service to address “poor compliance and poorly controlled diabetes”. She lives with multiple vascular complications including and recently has incurred incurred photo-coagulation treatment for bilateral retinopathy. She is accompanied by her daughter and a large list of medications. She has an aversion to taking medications and states that she had a “reaction” to taking diabetes medications 16 years ago (sulfonylurea). NP completed values assessment indicating that the patient places high value on her independence. In addition, a diabetes assessment identified that she scored highly for distress. Over the course of 2 weeks a trusting relationship was developed with the patient; all pharmacological treatment options were addressed. She was able to confer that she understood the relationship between her impending loss of independence and poorly controlled diabetes. With her daughter, a 3-month plan was established. Insulin was started with follow-up titration with the aid of community nursing service. Further, coordination with home community pharmacy review was established. These steps were aligned with the patient's goals to have continued interaction with her grandchildren and maintain her independence. Since her recent treatment for retinopathy and with insulin she has reengaging with her general practitioner (GP). Six-month progress review revealed no reoccurring urinary tract infections; feeling better and with “more energy”; increased interaction socially; reduced diabetes distress score and realization that insulin administration was easier than that she had anticipated.

*Critical/Emancipatory*. As an NP the practice of assessing patient adherence and prescribing, monitoring, and titrating medications has led to critically oriented change. Box 1 provides an exemplar of NP instigation and advocacy for the therapeutic and clinical feasible of a combination of insulin and glucagon like peptide 1 analogue (GLP-1) for a cohort of patients with type 2 diabetes. In addition, the identified need to provide primary care practices support in the initiation and titration of injectable treatments is being addressed through a collaborative forum with a Commonwealth supported primary care organization [57].

#### 3.3.2. Box 1

Nurse Practitioner Role; initiation, monitoring and facilitation of GLP-1 analogue and insulin combination therapy.

*Background*. For Tasmanians with type 2 diabetes (T2DM) sustaining a target HbA1c of 7% or under is less likely to be achieved than in any other state in Australia [64]. Most people with T2DM referred from primary care to a speciality endocrinology service in the state of Tasmania meet the criteria of nontarget glycaemic control and evidence of vascular complications. The majority of patients require high doses of insulin per body weight (>1 unit/kg), have sustained poor glycaemic control, and are obese with sequential multiple morbidities.

Options for glycemic therapy for insulin resistance and requiring patients;

- Insulin optimization, greater risk for hypoglycemia and weight gain, possibly increased cardiac mortality and further patient despondency [53, 65–67].
- Bariatric surgery (metabolic-surgical procedures) [68] is level 1 evidence treatment for morbid obesity [69, 70]; in Tasmania, bariatric surgery is not a realistic available option for public patients.
- Glucagon like peptide-1 (GLP-1) analogue and short term clinical trials provide strong pharmacological rationale for the combined use of GLP-1 analogue with basal insulin. Therapeutic efficacy is demonstrated through reducing exogenous insulin requirements, weight maintenance or loss, ability to target both fasting, and postprandial hyperglycemia and decreased risk of hypoglycemia [71–73]. Efficacy of GLP-1 analogue and insulin combination is superior in reducing weight and HbA1C% when comparing to insulin alone [71–73]. To date, North American and European regulatory agencies have approved the combined use of insulin and GLP-1 analogue on the basis of the efficacy and safety profile of their concomitant use in T2DM. In Australia, whilst this therapeutic combination is approved by the Therapeutics Goods Administration it is not subsidized by the national pharmaceutical benefits scheme [74]. This has resulted in an unaffordable therapeutic option for insulin requiring T2DM patients whose control is not to target. For endocrinology teams and patients, the context of knowing best practice options unobtainable due to costing regulation resulted in clinical dissonance.

*Aim*. The aim is to investigate the following questions.

1. What impact does the role of a Nurse Practitioner have on the addressing the issue of limited affordable therapeutic options for people with type 2 diabetes highly resistant, poor glycaemic control, living with multiple morbidities and obese?
2. How does critical reflexivity potentially enhance care?

*Method*. Reflexivity or reflection in action [75] goes beyond the usual introspection of (reflection on action) where consideration extends to the social and political context in which practice takes place. As a method of research in a clinical setting, critical reflexivity ensures a dynamic communicative partnership between researcher and practitioner [7]. For the purpose of this research, the “method of inquiry” developed by Kim et al. [44, 76, 77] was selected as a framework; it is founded upon the ideas of action science, reflective practice, and critical philosophy [37].

*Results*. Over a 3-year period the CRI method ensured positive despite being limited outcomes. NP Advocacy resulted in the State-Wide Drug Therapeutic Committee to afford 100 Tasmanian endocrine patients the combination of GLP-1 analogue and insulin. NP facilitation, coordination, and practice in pharmacological prescribing and monitoring of GLP-1 analogues have demonstrated a reduction in metabolic, biomedical, and physical markers in many recipients; also self-reported patient satisfactory in those afforded these pharm combinations [78]. To date this information has not led to the increase in placements in the state funded program. There continues to be a number of patients who could benefit from this combination therapy; an Economic Analyst has been approached to support further advocacy and practice research [78]. The assumption of increased indirect health care costs for the State Government due to increasing comorbidities will be a focus of this work.

*Conclusion*. Dissonance has been heightened for clinicians/practitioners acutely aware of the patient frustrations and struggle with insulin resistance and potentially dichotomous therapeutic outcome of optimizing insulin as a therapeutic treatment. The alignment of NP advance practice role and process of reflexivity, combined with the provision of high quality care, has facilitated improved therapeutic options for patients in Tasmania living with type 2 diabetes. Further advocacy is being undertaken through discussion for the development of economic rationale paper.

### 3.4. Vulnerable Populations

#### 3.4.1. Exemplar 1

*Description*. Vulnerable populations can be defined broadly to include any individual, group, or community whose circumstances present barriers to obtain or understand health information or to access health resources [58]. For patients living with mental illness their risk for diabetes is increased 3-fold from the general population largely as a consequence of lower socioeconomic status and the use of psychotropic medications [54]. This is particularly prevalent for people diagnosed with schizophrenia and taking second generation antipsychotic medication. In addition, this vulnerable population have a 2-fold increase in mortality from cardiovascular disease when compared to the general population [59].

*Reflection*. It was observed that a larger number of nonattendances for those referred to the specialist clinic were related to people who also had schizophrenia listed as a condition. NP initiated communication and a collaborative metabolic clinic with the local mental health clozapine service. This clinic has provided the integration of a metabolic screening pathway and access for metabolic assessment and counseling, in addition, provision of training of mental health team and case workers in decreasing patient's risk of diabetes.

*Critical/Emancipatory*. A 12-month pathology audit assessing the efficacy of metabolic screening showed a 2-fold increase in screening tests performed by clozapine clinic psychiatrists. The NP initiated a number of measures, pathways, and coordination with patient's general practitioners. In the case where a patient did not have a GP, the NP initiated treatment and coordinated care responsive to the person's metabolic syndrome and diabetes.

#### 3.4.2. Exemplar 2 (Box 2)

*Description*. A keen clinical observation and inquiry led one NP to children at risk for and living with congenital deformities. It was observed that too many women with pregnancies complicated by diabetes seemed to have adverse outcomes. Whilst evidence base was known, shortages in medical physicians expertise in diabetes management meant delayed care provision [11]. In addition, maternity services were contracted out to private organizations; thus screening procedures across the region were inconsistent. At the time there was no evidence to support the belief of paucity in both screening and management of diabetes in pregnancy. A clinical audit was undertaken to describe the “state of play.” In preparing for the audit and its potential results it was imperative for the NP to manage relationships with key stakeholders as many players were involved across several organizations.

*Reflection*. The NP noted that literature provides clear evidenced based guidelines in the provision of care of women with diabetes [60]. In addition, it is recognized that women within rural regional areas have an increased risk of adverse outcomes [60, 61]. It was observed that some of the care provided in metropolitan areas by medical practitioners could actually be performed by others holding competencies and skilled in diabetes and its management. It was recognized that new models of health delivery needed to be considered for rural regional areas to support the implementation of this level of care.

*Critical/Emancipatory*. With a role that encompasses both advanced assessment and treatment skills coupled with proficiencies in research, the NP role was well equipped to identify and manage stakeholder involvement while undertaking a quality improvement activity [27]. The audit supported by differing CEOs identified paucity and inconsistencies in screening and management which was presented to all key stakeholders [11]. A working party was established and led by the NP to develop a systemized model of care that supported screening and prompt early treatment as required. This also included identifying the potential risks of earlier but timelier commencement of insulin by diabetes educators. An interdisciplinary and systematic model of care was established and evaluated. The outcomes of the research project, including the reduction in adverse neonatal outcomes and cost associated to these, were promoted as an effective model of care for rural regional areas (Box 2).

#### 3.4.3. Box 2

Nurse Practitioner Role; translating diabetes in pregnancy research into everyday practice through facilitation and monitoring of a model of care in a rural regional area.

*Background*. For rural and regional Australian women with pregnancies complicated by diabetes the risk of complications is increased as a result of reduced access to specialist care and diabetes self-management education [61]. Socioeconomic status (derived from IRSAD) indicated 99% of women resided in areas which were among the four lowest deciles for socioeconomic deprivation in Australia [79]. On arrival to the region the NP (a candidate at the time) noted that access to screening, education, and treatment for women with diabetes in pregnancy (particularly GDM) was not consistent. Anecdotal evidence suggested higher incidence of complicated pregnancies and/or adverse effects to the infants, including congenital abnormalities, macrosomia, hypoglycemia, and birth injuries [60, 80]. Discussions with key stakeholders did not create any change for a number of reasons. There was uncertainty that there was a problem, changes to present services involved a number of differing facilities (some public and others private), and there was fear around the impact any changes would have on some physicians as this rural regional area was difficult to recruit to [11]. The NP candidate undertook discussing the concept of a quality improvement activity with CEOs of maternity services. This was approved and undertaken in the form of a clinical audit. It aimed to describe the current status of screening and care provision in the region for women with GDM, and the effects of maternal diabetes on the fetus, neonate, and mother.

*Aim*. The aim is to investigate the following questions.

1. What impact does the role of a Nurse Practitioner have on addressing models of care in rural regional areas?
2. How does critical reflexivity enhance care?

*Method*. Critical reflectivity goes beyond reflection of practice and encourages the potential researcher to explore their own practice to gain and advocate on new found knowledge. Freshwater and Rolfe (2001) indicated that it needs to “encompass a fully integrated practitioner who is immersed in both nursing and research as two aspects of the same role” [37]. This methodology fits perfectly with the NP role, a practitioner who has been trained at an advanced level and who comprehends and incorporates research into all spectrums of their role. The reflective/reflectivity cycle used in critical reflectivity encourages the modification of responses in the light of immediate feedback; this was used constantly during the information gathering/planning stages (particularly with key stakeholders and CEOs). Further, the cycle was used in personal reflective evaluation of the NPs own interventions as part of an ongoing sequence of actions and evaluation of those actions; within the led up to and following a small scale research project for diabetes in pregnancy.

*Results*. Over a 6-year period the critical reflectivity method gained positive outcomes in terms of reduction in adverse health outcomes, cohesiveness, and a systemized approach to care that encouraged interdisciplinary involvement. The small research project saw screening for GDM cases become universal compared to 33% of pregnancies preintervention, referrals to medical physicians significantly reduce (47.8% versus 15.2%, *P* < 0.0001), increased prompt treatment with insulin as required by CDEs, and a 24% risk reduction in adverse neonatal outcomes in all pregnancies [11]. In the GDM only group, the reduction postintervention compared to preintervention in adverse neonatal outcomes was 40% [11]. This population of patients continues to benefit from this project with the NP leading to a DIP Working Party where processes and systems within the clinics continue to be monitored for inclusion of new evidence based practice as it arises. Further, through the development of a close interdisciplinary approach incorporating the obstetrician, dietitian, Credentialed Diabetes Educator (nurse), and midwife.

*Conclusion*. The Nurse Practitioner role can support and drive changes to health models that create positive health outcomes for infants born to women with diabetes. A strength of the DIP NP led model is its systematic approach that incorporates contemporary evidence to ensure safe and quality care to deal with the a paucity of medical specialist services in rural areas. The model integrated material from international research, Australian guidelines and local clinicians; ensuring the model was both evidence-based but relevant to the local healthcare context [11]. The alignment of the NP advanced practice role and the process of critical reflexivity and provision of high quality care has facilitated a clear systematic integrated care pathway. This will support the continued provision of evidence based care regardless of the health professional changeover, as is common in rural regions.

### 3.5. Leadership 

#### 3.5.1. Exemplar 1

*Description*. Often health care pathways are developed with good intentions; however, due to lack of complete stakeholder involvement full integration is frequently missed [62]. Through differing venues one NP noted that the primary care provider “Medicare Local” intended to create pathways for GPs to assess and manage diabetes. The NP aware of the gaps in transition across the care organizations with patients with complex health care needs moving in and out of hospital identified this as an opportunity to support an in-depth consultative process, promoting the NP as a role to support timely appropriate care. Previous local pathways had not involved all relevant stakeholders and thus did not necessarily meet identified gaps or support engagement.

*Reflection*. The alignment of the NP clinical expertise, role in care coordination, and leadership qualities has the potential to strengthen the development of health care pathways while ensuring inclusion of evidenced based. Inappropriate referrals easily managed by the GP and diabetes educator often blocked specialist clinics increasing waiting lists [50]. Alternatively, some patients with complex needs failed to be referred in a timely manner to reduce impact of complications. The NP considered how the NP role “value added” to this process to ensure incorporation of evidenced based care and increased access? In addition, the main issues faced by patients using these health services and how the pathways would remedy this?

*Critical/Emancipatory*. Critically orientated changed occurred as a result of the NP leadership skills. The NP used this opportunity to reignite Diabetes Key Leaders Group utilizing leadership qualities to meet with key stakeholders individually and discuss the benefits of developing partnerships. This became a platform of relevant senior state-wide health professionals/managers for diabetes to meet and create opportunities; the Medicare Local “Health Pathway” developer was invited to present to the group. In the 12 months following, the NP was invited onto a membership to review the pathways [63]. To enhance the process the two NPs encouraged the mapping of diabetes services across the state by the peer group. This has led to NP coordination of a meeting to scope service models in the state for government funded and not for profit organization. This scoping utilized to identify gaps and opportunities so that these are fed back into pathway development.

#### 3.5.2. Exemplar 2

*Descriptive*. Leadership roles within the state are often held by medical officers. The NP role in this state of Australia was only more recently implemented and was generally an unknown entity [16]. Often decisions are made that alter health care service delivery that does not recognize the potential of alternative safe models of care with existing resources through careful coordination.

*Reflective*. Membership to the expert group that would drive health changes in the state was identified as an opportunity for raising the profile of NPs. One NP was nominated to the group by the Minister of Health following application. As the expert group met and as meetings progressed the Chair acknowledged their strong support of the NP role and its contribution, feedback and comments by the NP were well received by committee members; the NP focused on the use of this positive discourse to further identify the NP as an expert resource that is able to contribute to policy development at a high level. It was important to identify what this opportunity would bring to NPs. The NP reflected on their actions and scrutinize “what had just happened” in each context with a desire to improve leadership skills—a deep inward gaze into every interaction whether it be in meetings or presentations and/or other interaction as an NP.

*Critical/Emancipatory*. Over the course of 18 months the positive contribution of the NP was communicated in various forms and including the Minister of Health, identified as a key player with strong leadership skills. The NP was selected to represent the advisory group at several major meetings including a “Health Commission” and “Strategic Planning Day” of the Governing Councils. This exposure led to an invitation to present at a Tas Health Conference which was predominant medical conference showcasing medical research in the state, the NP presented on the nature of diabetes in the state and NP research. In addition, the NP contributed to the making of a small report played on the Australian Broadcasting Corporation TV to promote NP roles. As an NP the sharing of competencies in leadership, care coordination and research within a peak expert body have led to critically oriented change. Other NPs were sought out for the next Tas Health Conference; the NP is recognized as a key player in research and care redesign.

## 4. Conclusion 

The NP application of CRI resulted in an inductive and reflective view of practice that led to facilitating and providing evidence for critically orientated practice change. CRI as a methodical process provides direction for the NP to engage in each of the CRI phases: descriptive, reflective, and the critical emancipatory. The results of this research demonstrate the utility of CRI in the NP generation of “products” (under each phase) responsive to the authors' NP practice. These products have served as foundational components in the articulation of NP practice value for their patients and health care systems that they serve. For the purpose of this research the NPs have demonstrated an alignment of their practice to health care values of Specialised Care Access, Complications and Diagnostics Interventions, Pharmaceutical Treatment, Vulnerable Populations, and Leadership. Critical oriented change was demonstrated as integral to the NP's practice and the results exemplify the NP's capacity to provide substantial change in the provision of safe and timely health care when addressing deficiencies/gaps in care provision. It is anticipated that the application of CRI demonstrated will inspire other Australian and/or International NPs in the articulation of the value their NP practice has in the context of economic nationalization. Potentially, the application of CRI by NPs, may also serve to influence and expedite policy decisions and subsequent funding models to support the viability of current and future NP practice.

## Figures and Tables

**Table 1:** Critical Reflective Inquiry: Nurse Practitioner Integration.

CRI process	Descriptive phase	Reflective phase	Critical/emancipatory phase
Prompt questions	(i) What are the common demographics and contributing factors for patient referrals/episodes? (ii) What are the reasons for patients seeking care? (iii) Which patient groups are denied timely access to safe health care and does this lead to deterioration of condition? (iv) What are the prominent NP interventions that are needed to address improvement in health outcomes? (v) What barriers exist for patients in improving health outcomes?	(i) What contextual health care issues (poor access to speciality care, high risk for hospitalization, need for improved coordination among providers) are or are not being addressed? (ii) What further empirical knowledge (e.g., clinical guidelines, pathways) could be applied to patient episodes? (iii) Are there ethical and value standards that are incongruent in the treatment of patients? (iv) What resources and NP interventions could address broader issues of patient referrals/episodes?	(i) Is there a need to change how NP practice is meeting contextual health issues? (ii) What capacity is there for change in practice (and/or innovation to practice?) (iii) What is the feasibility to changes in practice? (iv) What are the benefits and risks for change? (v) Who is the change likely to impact? (vi) How would impact to patient care be measured? (vii) What value would this impact have on individual patient care and health systems?

Products	Descriptive narratives, reflective data collection and includes case studies and clinical audits.	Examination of personal beliefs, assumptions, and knowledge as aligned and tested with existing scientific knowledge, analysed in terms of ethical or valued standards.	Oriented towards changing practice through recognition of discrepancies in practice and best practice, a critique of distortions, inconsistencies, and incongruences between values/beliefs and practice.

Value	Definition of common themes that are congruent with health care system needs for reform reflected in clinical practice. Objective and qualitative data collection to formulate indicators for change.	Alignment of NP values to practice of complex care cases/scenarios and in comparison to systematic incongruences in the application of practice.	Innovations to clinical practice that address the systemic indicates for poor health outcomes or inequitable healthcare provision.

**Table 2:** Summation of results: practice themes, NP interventions and innovations, and value to health care systems.

Practice themes	NP interventions and innovations	Value to health care systems
Specialized care access	(i) Development of clinics responsive to service gaps; for example, rapid response and consistent care/systematic clinics	(i) Improved access to speciality care (ii) Aversion of emergency department and hospital admission (iii) Decreased waiting times for speciality care clinics (iv) Reduction on adverse health outcomes

Diagnostic and complications	(i) Clinical expertise and clinical alignment in advance practice interventions in the review of people with complex healthcare issues and the interplay of complications and comorbidities	(i) Concerted and patient specific care plans that address multifactorial factors that contribute to a patient poor health outcomes such as deteriorating glycaemic control, increased infections, and risk for mental health status (ii) Detection of other conditions impacting on health care not previously identified

Pharmaceutical treatment	(i) Timely and functional review and prescribing, titration and monitoring of medication (ii) Advocacy and development of pharmaco-therapeutic options for poorly controlled patients living with multiple complications and comorbidities	(i) Improvement in patient knowledge and efficacy in medication management (ii) Responsive communication channels developed for patients to engage with NP to obtain individualized and achievable biomedical, metabolic, and glycaemic targets in effective time frame (iii) Advocacy and coordination of innovative treatment options for patients with demonstrable improvements compared to status quo treatment

Vulnerable populations	(i) Targeted intervention for vulnerable populations, including delivery of multidisciplinary clinics and changes in models of care to address poor health outcomes	(i) Significant reduction in congenital malformations pre and postdelivery of GDM care model of care (ii) Quantifiable improvement in screening and treatment of metabolic syndrome and diabetes for patients living with schizophrenia

Leadership	(i) NP Participation, representation, and leadership roles in strategic initiatives in forming alliances between tiers of health care systems	(i) Contribute to development of state-wide referral pathways for improving care for patients living with diabetes (ii) NP committee participation for health care reform has provided a voice for the role and demonstration of the clinical leadership
